# Insight into diatom frustule structures using various imaging techniques

**DOI:** 10.1038/s41598-021-94069-9

**Published:** 2021-07-15

**Authors:** Izabela Zgłobicka, Jürgen Gluch, Zhongquan Liao, Stephan Werner, Peter Guttmann, Qiong Li, Piotr Bazarnik, Tomasz Plocinski, Andrzej Witkowski, Krzysztof J. Kurzydlowski

**Affiliations:** 1 Faculty of Mechanical Engineering, Bialystok University of Technology, Białystok, Poland; 2 Fraunhofer Institute for Ceramic Technologies and Systems (IKTS), Dresden, Germany; 3 X-Ray Microscopy Department, Helmholtz-Zentrum Berlin (HZB), Berlin, Germany; 4 Faculty of Materials Science and Engineering, Warsaw University of Technology, Warsaw, Poland; 5 Institute of Marine and Environmental Sciences, University of Szczecin, Szczecin, Poland

**Keywords:** Nanoscale materials, Structural materials, Techniques and instrumentation, Mechanical engineering

## Abstract

The diatom shell is an example of complex siliceous structure which is a suitable model to demonstrate the process of digging into the third dimension using modern visualization techniques. This paper demonstrates importance of a comprehensive multi-length scale approach to the bio-structures/materials with the usage of state-of-the-art imaging techniques. Imaging of diatoms applying visible light, electron and X-ray microscopy provide a deeper insight into the morphology of their frustules.

## Introduction

Nature is a source of complex materials which possess a wide range of complementary or synergistic properties. Multi-scale structures in biological organisms determine their behaviour, simultaneously this elegant perfection makes scientists awestruck. Beyond delight, engineers make attempts to emulate solutions as well as designs invented by Nature in man-made innovations. A range of technological advances inspired by living organisms also known as biomimicry is broad and become widespread^1–9^. There is no doubt that solutions presented by Nature are well ahead of every engineering material. The standard approach in materials science as well as mechanical engineering is to conduct insightful observations of such materials/structures. Nevertheless, the key for understanding the structures of natural organisms is to develop new but firstly adapt already known methods for high resolution imaging.

One group of ubiquitous microorganisms demonstrating the diversity in the morphology and structure, on various scales, are diatoms^10–13^. The morphology of their siliceous shells (= frustules) is highly elaborated. Substantially, many details responsible for the properties of these structures, e.g. mechanical ones, may be observed by high-resolution imaging^14–16^. According to the literature, the mechanical properties of the diatom frustule depends on the location. Experiments conducted on *Navicula pelliculosa* showed that the highest values of the elastic and hardness modulus, respectively up to hundreds of GPa and up to 12 GPa, have been obtained at the central part of the frustule^11^. These results have been confirmed by Subhash et al.^17^ conducting investigations of hardness and fracture modes of the frustule of *Coscinodiscus concinnus*, higher values have been obtained in the central nodule which is solid. In addition to this, the fracture resistance also depends on the size of the diatoms^18^. This values are regarded as remarkably high, for shells made of relatively soft bio-silica^19^ and discussed in terms of guarding cells inside frustules against predators^18^.

Advances in studies of diatoms are dating back until the early nineteenth century^20^. Beginnings were associated with hand illustrations, based on light microscopy observations. The further development of this technique, lead to the possibility to record images of observed samples using a camera. Further improvement of the research methods include application of electron microscopy (EM), both scanning and transmission electron microscopy (SEM, TEM)^10^. Electron microscopy allows to examine the structures of a single shell with much greater detail. Nevertheless, progress in knowledge of the construction, based on EM, is always a matter of good luck—as a consequence of the sample position arrangement. Authors made also attempts of various sample preparation procedures relying on putting a single frustule on a stub. However, none of these approaches did allow to observe cross-sections of the natural structure. This limitation has been overcome by the Focused Ion Beam (FIB) technique. This technique is an option to provide information about internal structure by ion-based cutting of the sample. Several diatoms species have been investigated using FIB-SEM^14,21–24^. Non-destructive investigations of the structure can be achieved by nano X-ray computed tomography. A detailed study of frustule structure using advanced high-resolution imaging—comparison of FIB and nano-XCT—has been published^14^. Nano-XCT allows to non-destructively obtain a 3D data set of the diatom structure with detailed morphological information. Tomographic data sets allow to take arbitrary virtual cross-sections through any region of the frustule, so this technique provides access to additional dimensions. In the aim to capture the finer details of the diatom frustules, synchrotron based soft X-ray nano-tomography can be used.

The aim of the presented paper is to show the advantage of using multi-length scale imaging techniques applied to get insight into biological structures on an example of diatom frustule. The paper shows examples of the images obtained with light microscopy (LM), electron microscopy (both scanning and transmission) and X-ray microscopy, each the resolution varying from standard to high.

**Figure 1:**
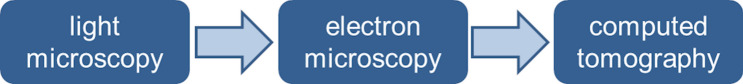
Flow diagram presenting the subsequent observations methods.

The novelty of the approach presented here is in the comprehensive multi-length scale description of the bio-artefacts in question allowing for holistic approach to their structure–functionality (Fig. 1).

## Results

**Figure 2:**
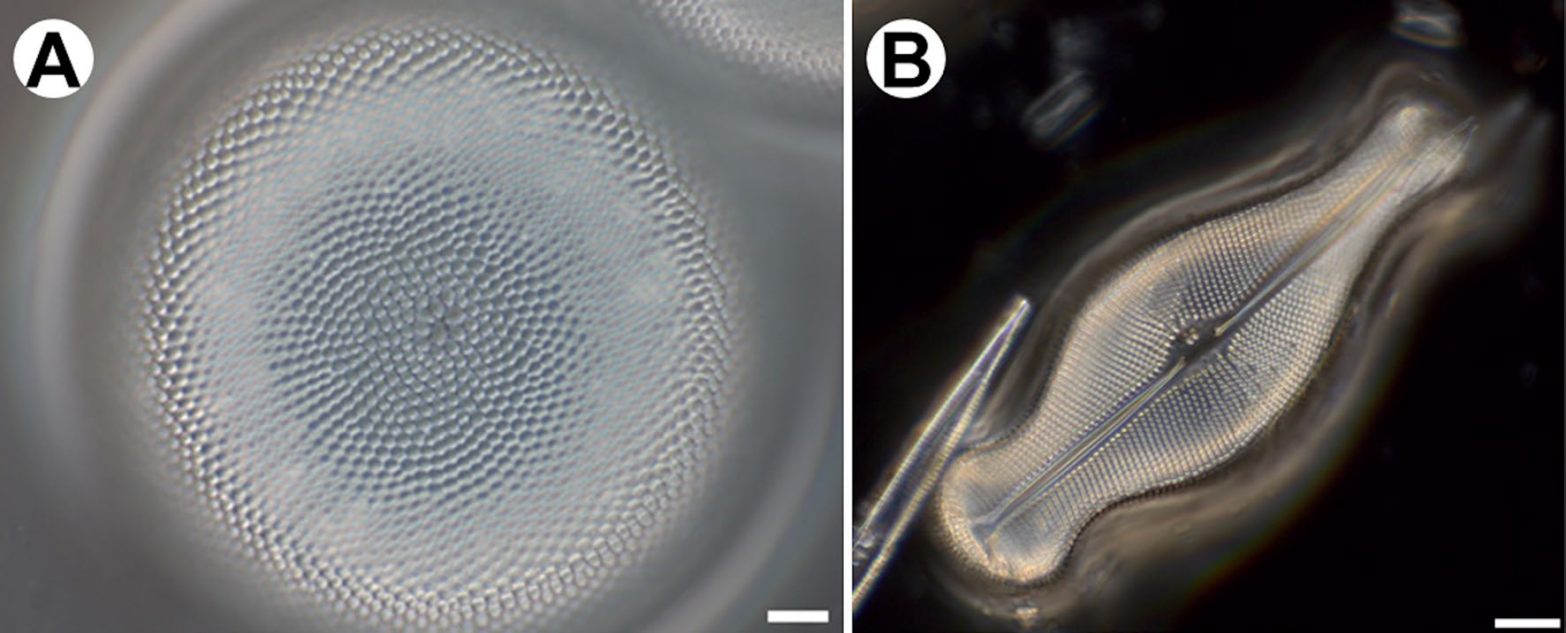
Light Microscopy (LM) images of (**A**) *Coscinodiscus* sp.; (**B**) *Didymosphenia geminata*. Scale bars: 10 μm.

Light microscopy (LM) images (Fig. 2) show the overall diatom frustule based on which the distinct shape (symmetry) as well as size (*Coscinodiscus* sp.—100 μm, *Didymosphenia geminata* sp.—100 μm) can be defined. The characteristic, periodic features occurred on the valve can be also distinguish. The *D. geminata* frustule has been imaged with the usage of the dark field microscopy which allow to visualize spacing of the shell.

**Figure 3:**
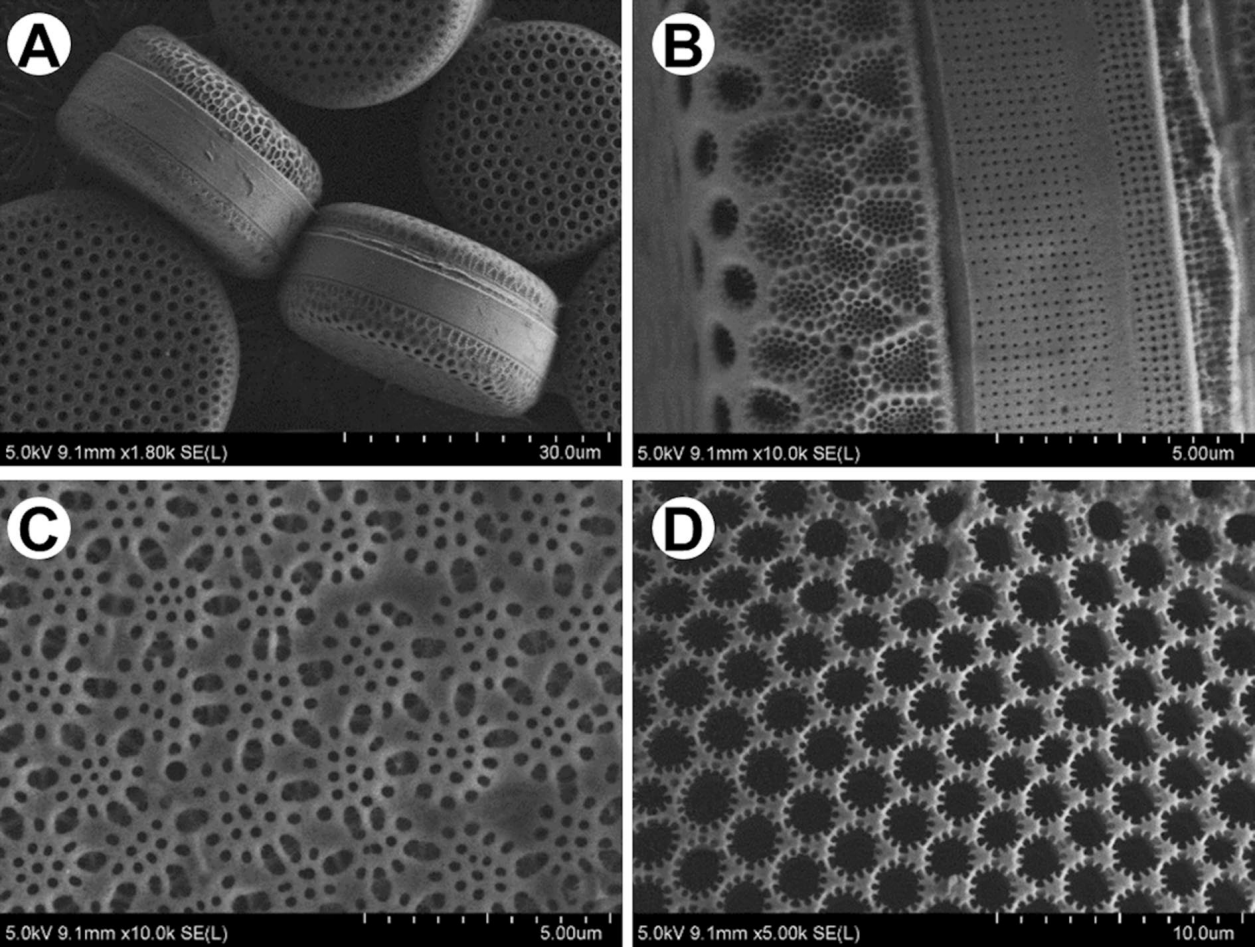
Scanning Electron Microscopy (SEM) images of (**A,B**) *Endyctia* sp. and (**C,D**) *Coscinodiscus* sp.: (**A**) valve and girdle view; (**B**) morphology from the girdle view; (**C**) outer side, (**D**) inner side of the valve.

The whole frustule, like in LM, can be also visualized with scanning electron microscopy (SEM). This technique gives a possibility to demonstrate the intricate, highly patterned silica shell, from both valve and girdle view (Fig. 3A,B). Furthermore, complex structure of the frustules is also well visible in a variety of the patterns depending on the surface—outer (Fig. 3C) and inner (Fig. 3D).

**Figure 4:**
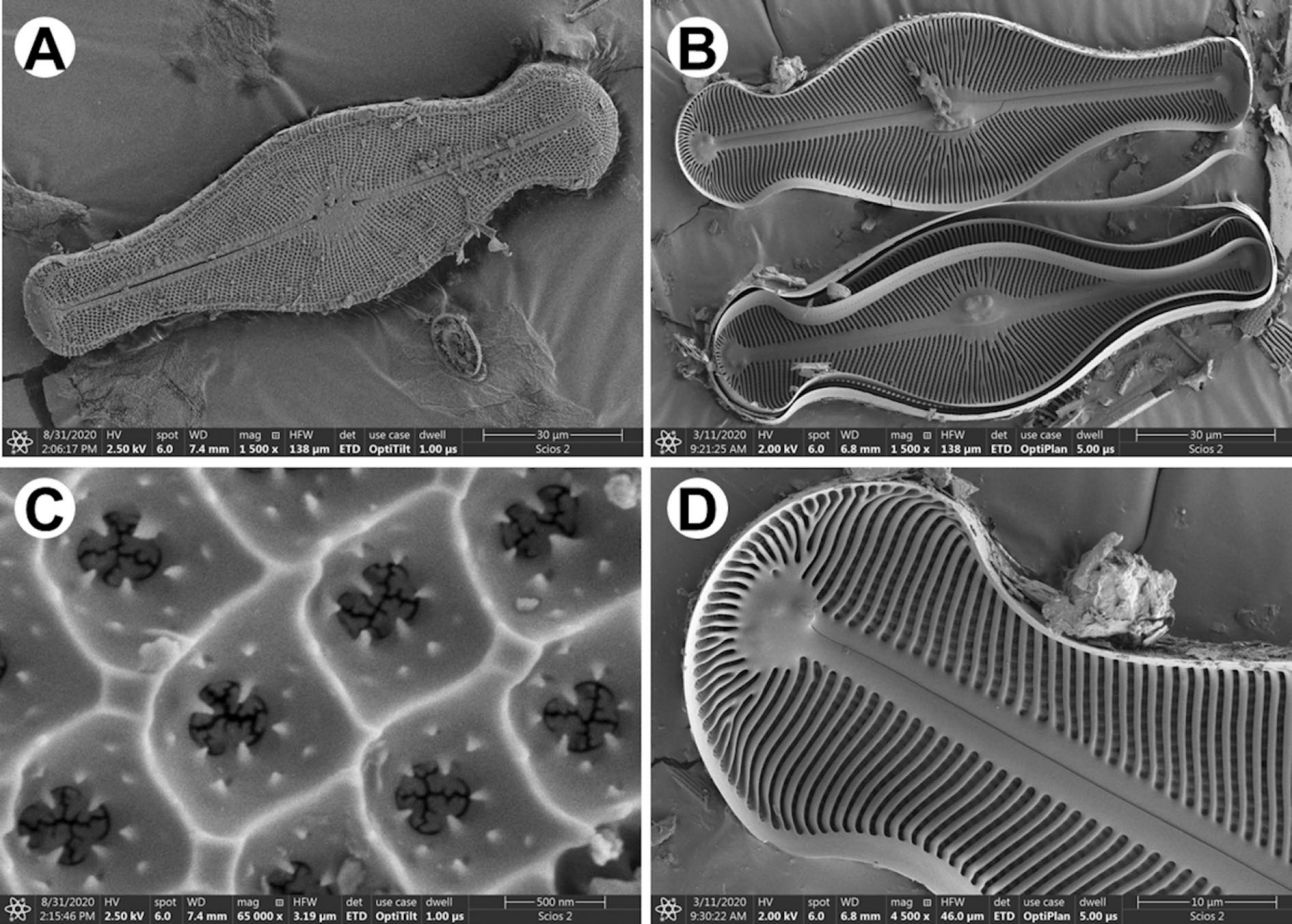
SEM images of *Didymosphenia geminata* frustule: (**A**) valve view; (**B**) inner side of the valve; (**C**) close up of the valve view—note the areolae openings; (**D**) magnification of the inner side of the valve—head pole.

Nevertheless, arrangement of the shell on the stub, without additional intervention, is an issue related to the drying process (Fig. 3A). The preparation procedure, which aim is to get rid of organic matter as well as impurities (Fig. 4A) embedded on the surface, may result in separation of the valves. It gives a possibility to observe interior of the shell (Fig. 4B,D).

Higher magnifications used during SEM observations allow to distinguish openings (Fig. 4C), which good examples are areoles in *Didymosphenia geminata*. Images present their funnel-shaped shape as well as roughly in the middle of the height of each funnel the horns around and a membrane at the bottom. SEM images allow to depict differences in appearance of outside (Fig. 4A) and inside (Fig. 4B) surface.

**Figure 5:**
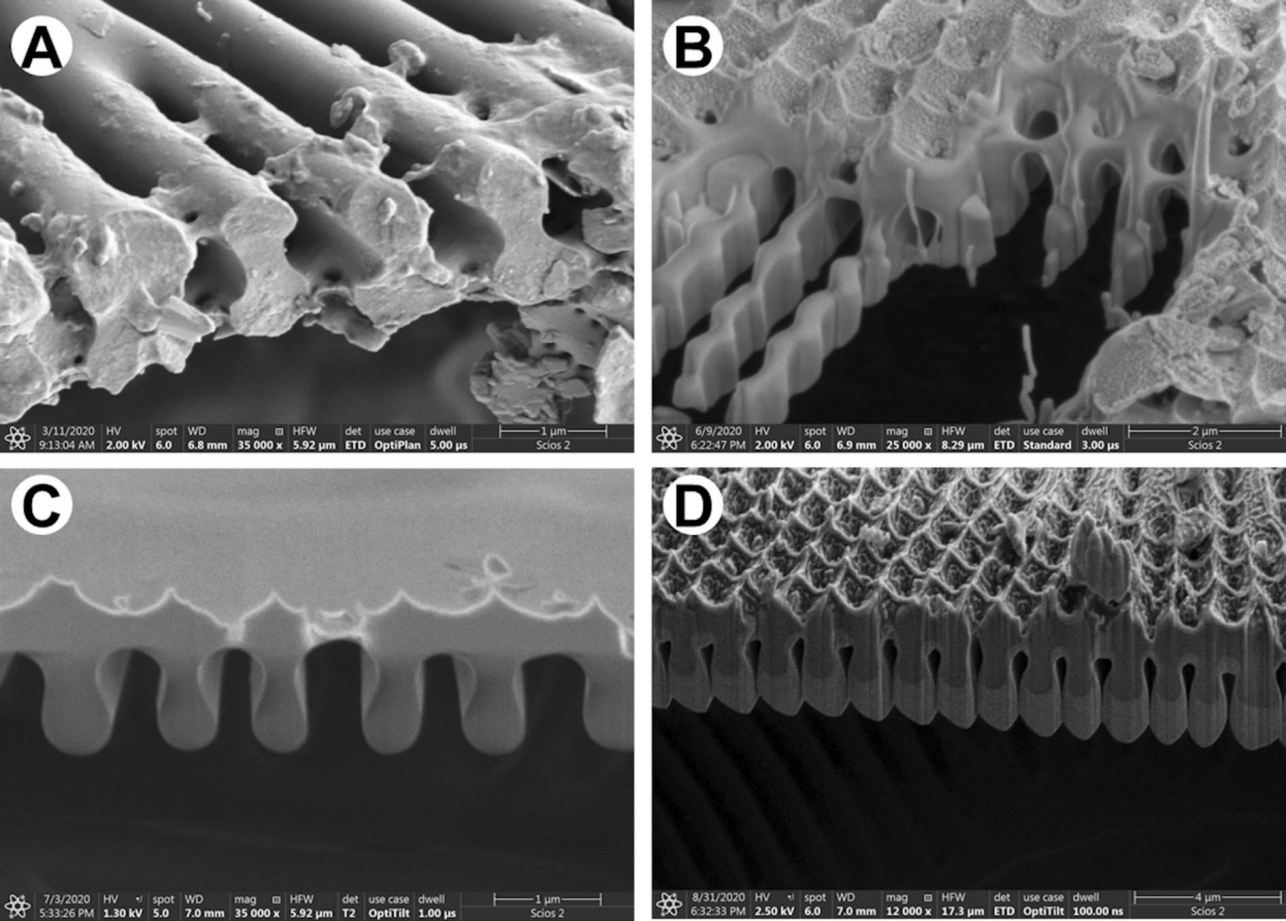
SEM images of cross-section of *D. geminata* valve: (**A**) naturally broken shell; (**B–D**) after FIB cut.

In must be noted that the internal structure is the best visible on the cross-section of the frustules (Fig. 5A,B). It can be obtained via usage of scanning electron microscopy–Focus Ion Beam (SEM–FIB). Based on images it can be concluded that exemplary openings in *D. geminata*, areolae, originate through a series of ribs and bridges which connect ribs between themselves (Fig. 5B). Additionally, the preparation procedure is very important due to the fact that delicate morphology (Fig. 5D) may be lost with the usage of the Tungsten layer (Fig. 5C).

**Figure 6:**
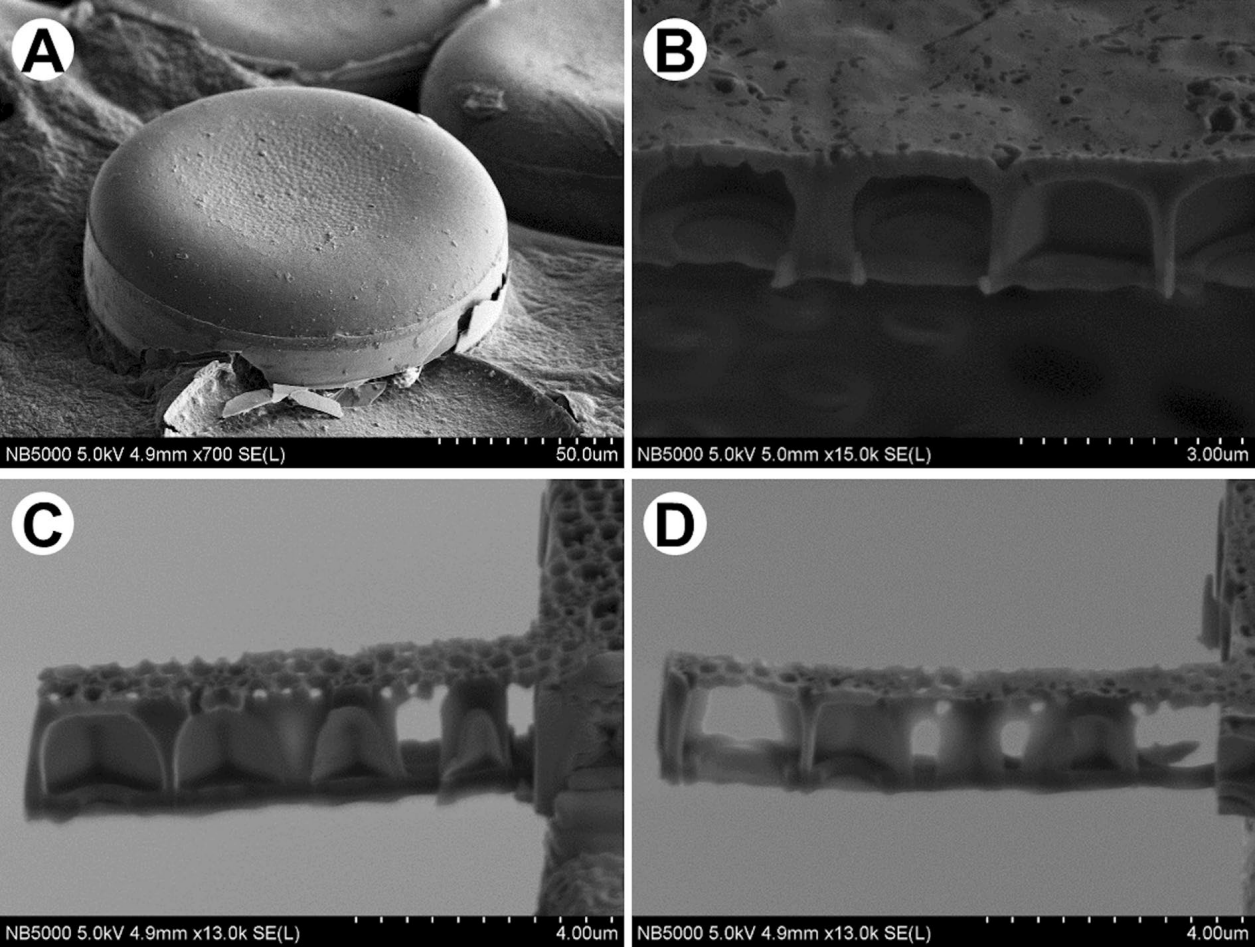
SEM images of *Coscinodiscus* sp.: (**A**) whole frustule; (**B**) cross-section of the shell; (**C–D**) samples prepared with ion beam for synchrotron observations.

Every diatom species is characterized by their own morphology and structure. Some of them, like i.e. *Coscinodiscus* sp. (Fig. 6A) presents hierarchical structure of the frustule. It is characterized by a different size of the pores, frequently from micrometers to nanometers (Fig. 6B–D). Except of the preparing cross-sections of various samples, FIB-SEM is very often used for sample preparation—lamellas (Fig. 6C,D)—for i.e. transmission electron microscopy (TEM) as well as computed tomography (CT).

**Figure 7:**
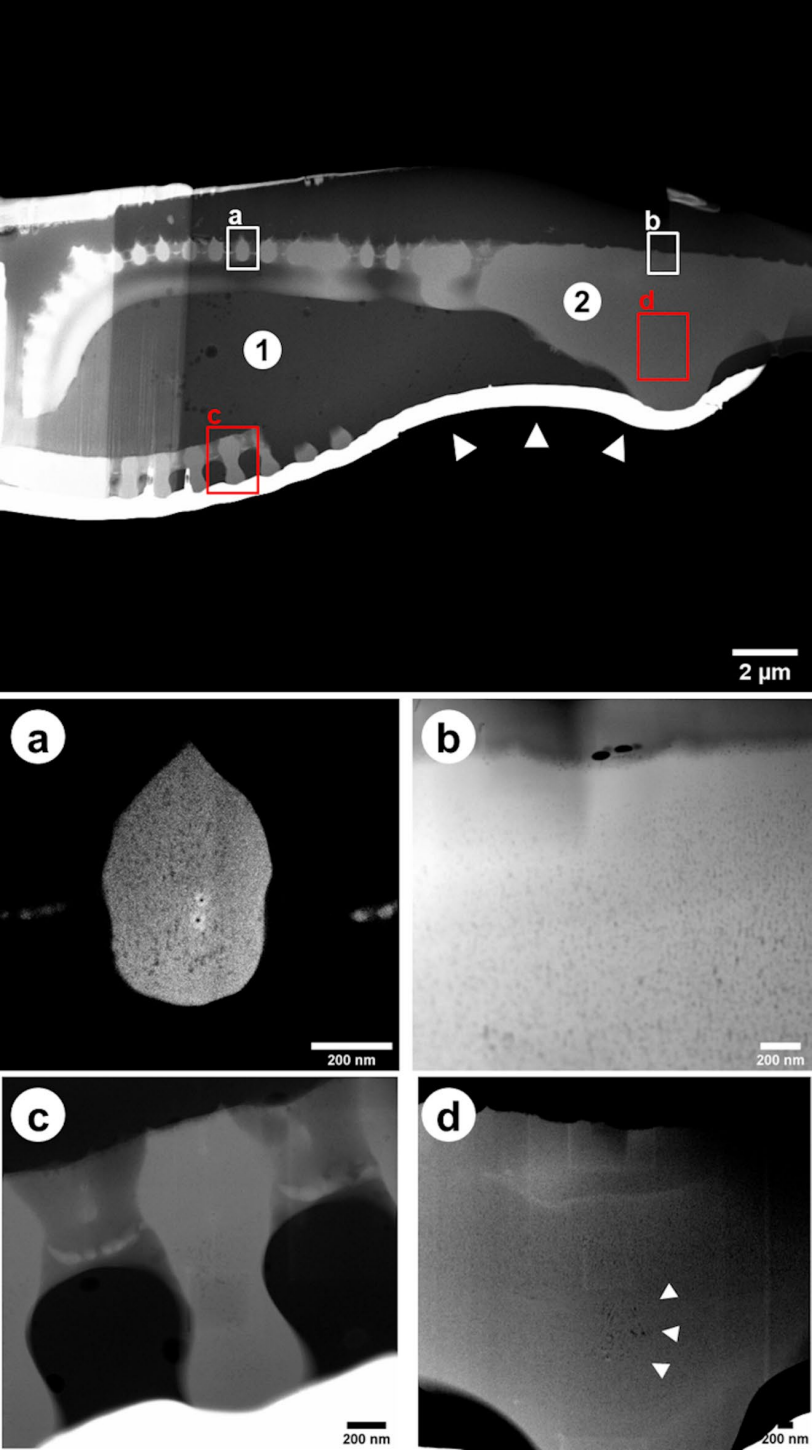
TEM dark field images. Cross-section of *Didymosphenia geminata* frustule: (1) epoxy filling, (2) frustule, white arrowheads: Pt protection layer; (**a**) single rib with increased contrast to visualize the pores; (**b**) central area with dense layer on outside; (**c**) other rib shows also uneven distribution of pores; (**d**) central area close to stigma with some bigger pores (white arrowheads).

Representation images of transmission electron microscopy of *D. geminata* frustule embedded in epoxy filling prepared using FIB in the SEM are presented in Fig. 7. Thin membranes with unique structure in between the openings (areolas) between the ribs can be observed (Fig. 7a). The thickness is about 50 nm. Based on obtained images of *D. geminata* (Fig. 7), it can be concluded that density in the frustule is not constant. The ribs of the frustule show an entire porous structure (Fig. 7a), whereas the central part shows a dense outer layer and a porous layer towards the inside of the frustule (Fig. 7b). Some of the ribs show uneven distribution of the pores (Fig. 7c). In the central region, next to stigma, bigger pores can be observed (Fig. 7d). The thickness of the layers depends on the location in the frustule. A dense layer can be up to 200 nm in thickness.

**Figure 8:**
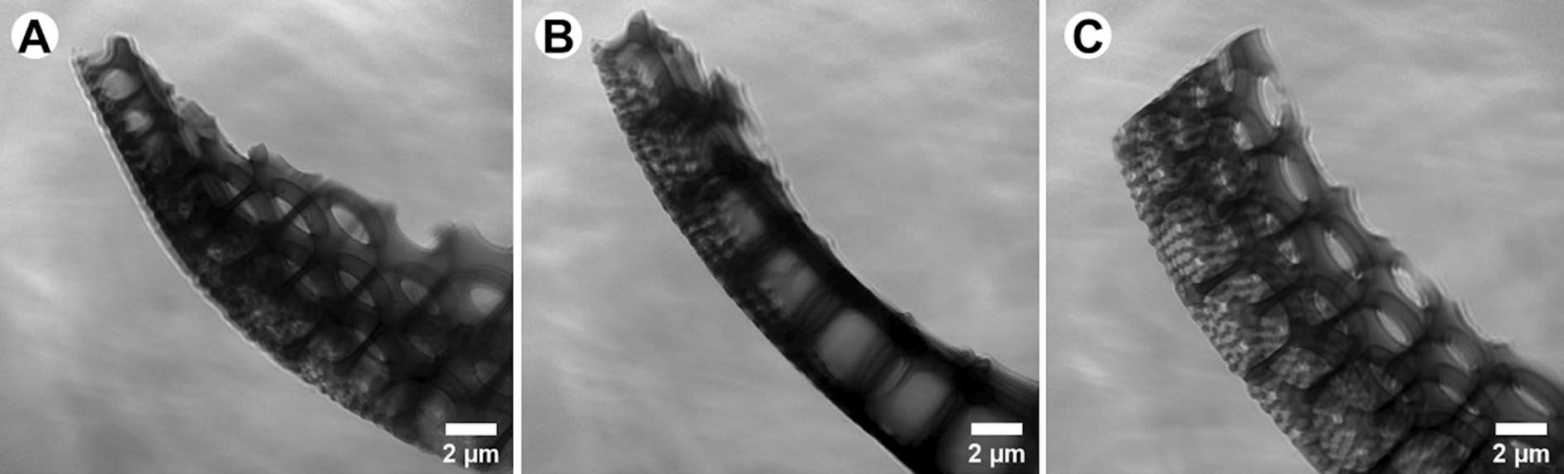
Images of diatoms frustule *Coscinodicus* sp. obtained by soft X-ray nano tomography investigations.

Visualization of the diatoms frustules structures can be also conducted with the full-field transmission X-ray microscope using synchrotron radiation. Soft X-ray nano-tomography experiments performed at the electron storage ring BESSY II result in a set of images which allow to prepare the 3D visualization of the investigated samples (Fig. 8).

**Figure 9:**
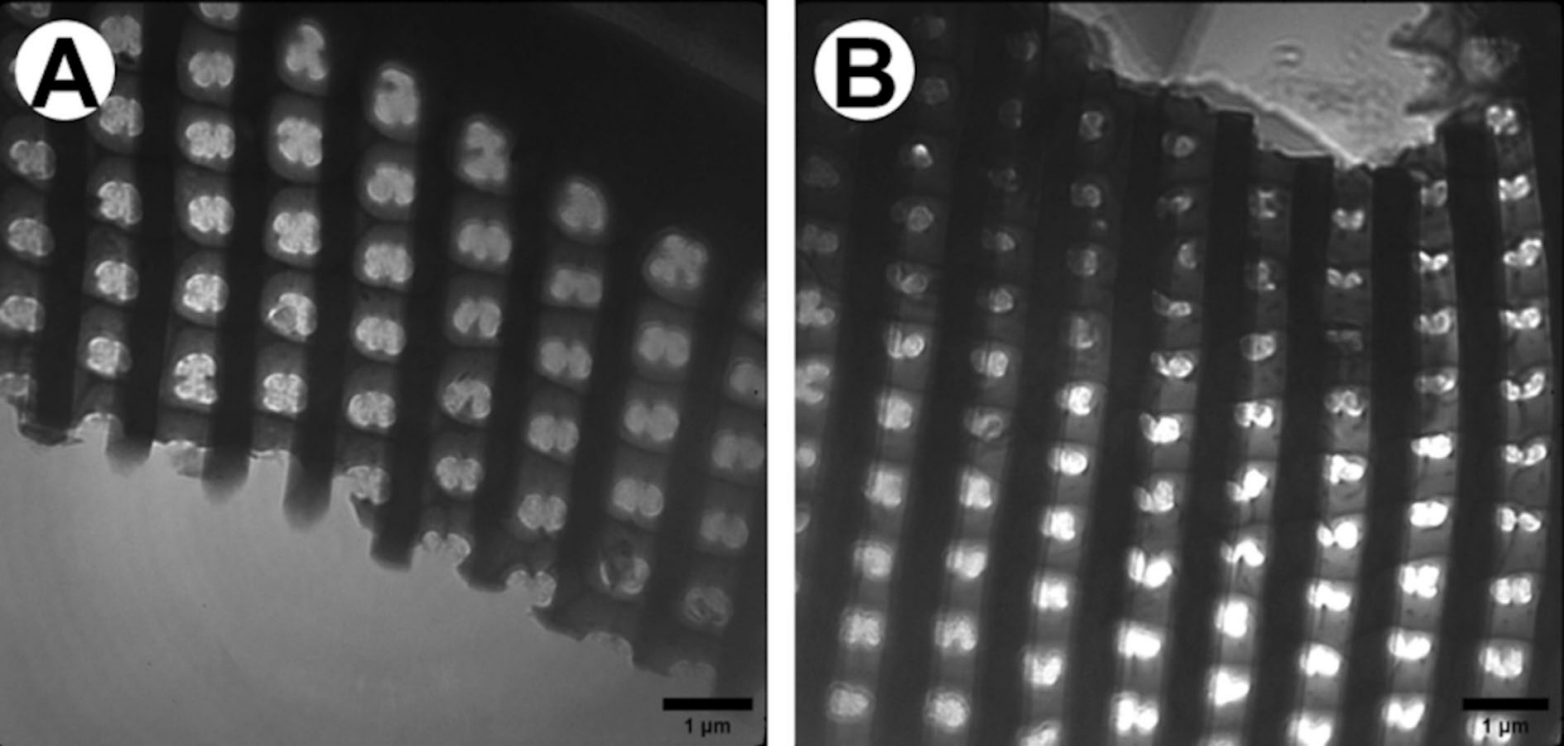
Comparison of the used techniques (**A**) TXM with synchrotron raditation and (**B**) TEM.

The soft X-ray nano-tomography resolution allows to distinguish the hierarchical architecture of the frustule (Fig. 8) as well as tiny structural elements (Fig. 9). This hierarchical architecture results from the various sizes of the holes from inner and outer side of the shell. Furthermore, the connection between subsequent layers also can be distinguished on the cross-section of the frustule.

The laboratory based nano X-ray computed tomography (nano-XCT) allows to non-destructively visualize the structure of diatom frustule. It must be noted that computed tomography allows to conduct visualization in two contrast modes—absorption (AC) and phase (PC) contrast.

**Figure 10:**
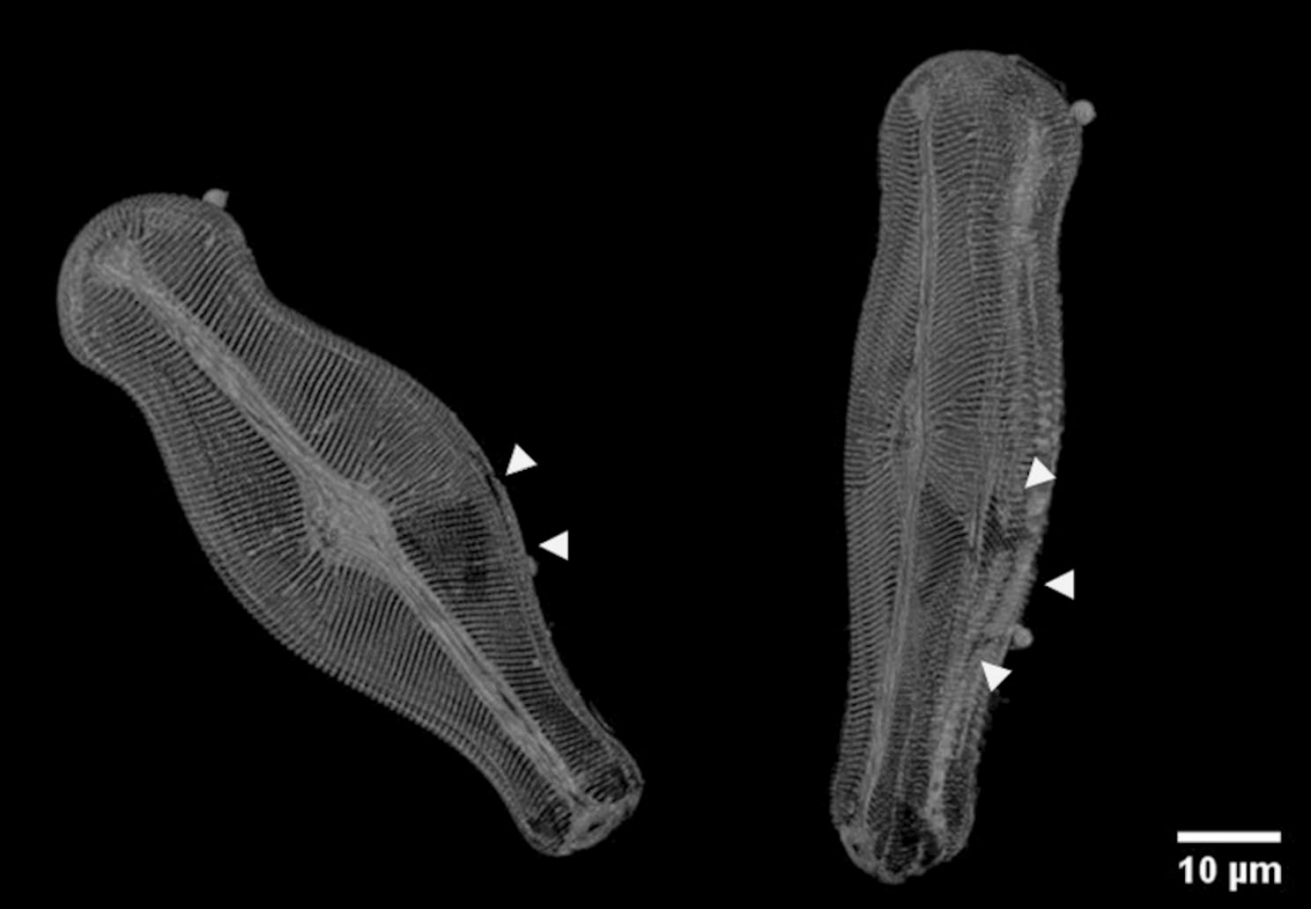
Visualization of *D. geminata* frustule obtained based on data from two separate tomography data sets: top and bottom of the frustule; arrowheads: voids due to the connection of two tomography data sets.

**Figure 11:**
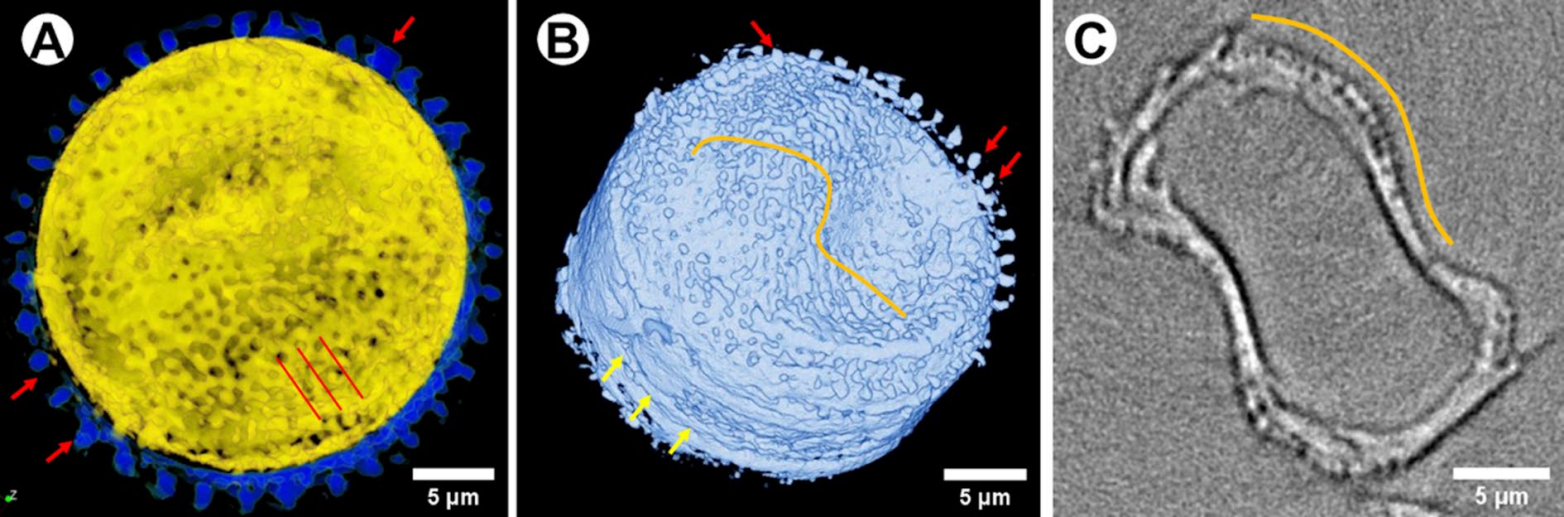
The *T. lacustris* imaged by nano-XCT. (**A**) Valve surface of *T. lacustris*, (**B**) the 3D rendering of the of *T. lacustris*, (**C**) oblique view of the tangentially undulate valve and pores on it. *Orange curve line* tangentially undulate valve face, *red arrows* a ring of marginal fultoportulae, *red straight lines* the coarse and organized striae, *yellow arrows* the girdle band.

The experiments conducted on exemplary diatom—*D. gemianata* because of the overall length of the shell (ca. 100 µm)—required to prepare two separate tomography data sets, which afterwards have been combined. The combination of two sets of data may results in unnecessary voids created during reconstruction (Fig. 10, white arrowheads). The visualization of the shell in case of diatoms with smaller frustule, like i.e. centric *Thalassiosira lacustris* (size ca. 25 µm) do not require such treatments and there are no concerns about discontinuities in the visualized 3D data (Fig. 11).

The visualization of the diatom *T. lacustris* allows to observe tangentially undulated valve faces, organized and coarse striae and a ring of marginal fultoportulae (Fig. 11).

## Discussion

The natural organisms like diatoms present seemingly simple, yet sophisticated structure which can be shown with the usage of various imaging techniques. It is essential to provide both, an entire view of the sample as well as accurately visualize the details.

The presented paper covers the results obtained for the selected diatoms. It must be noted that variety of diatom species (ca. 100,000–200,000^25,26^) gives wide possibilities for scientists for further investigations.

Based on the approach, the “work-flow” which covers the proper order of observations has been established. There is no doubt that within chosen ones should include the techniques which provides information about the entire structure as well as features at appropriate for them degree of accuracy.

The most common method based of studying diatoms in the past was Light Microscopy (LM). The images obtained in this way are widely used for determination of size as well as shape of the single frustule and are bases for their classification. The resolution of light microscopes, however, is insufficient to reveal details of the diatom frustules.

Scanning electron microscopy (SEM) offers higher magnification and depth of focus in comparison with LM. This technique allows to identify smaller structural elements in the architecture of frustules, like openings and what is important their curvature. Nowadays when combined with Focus Ion Beam options, it can also be used to reveal details which can be visualized only by sectioning of the diatoms. FIB makes a target preparation possible.

The characteristic features of the morphology revealed with the use of SEM–FIB include, at least: connections between outer and inner layers, degree of areolae closure. The FIB technique can be also used to prepare thin sections for transmission electron microscopy (TEM).

The newest technique used in study of bio-artefacts is Transmission X-ray Microscope (TXM). Unquestionable advantage of transmission mode is providing the detailed structural information with up to atomic resolution. Some of findings presented in this paper, like uneven porous structure of the ribs, are a new knowledge about diatom frustule and require further investigations. Such tiny elements may be observed due to the thickness of the investigated sample which has been translucent as well as low density of the material.

The 3-D nondestructive visualization of the sample can be realized with the use of computed tomography (CT). This technique gives wide possibilities of visualizations the whole frustule as well as small part of it. The undisputed advantage is that data obtained from computed tomography can be converted into 3D file and up-scaled for better visualization.

Additionally, based on data obtained during insight into biological structures, the calculations of characteristic features can be conducted. The comparison of all techniques allow to conclude that CT measurements, due to the possibility of evaluation of size and density of the openings, dimensions of ribs as well as depth of stigma are more accurate and easier to obtain. It must be noted that all these measurements can be conducted without destroying the investigated sample.

## Conclusions

The natural architected materials show great variety in shape, morphology and structure, which makes it difficult to obtain a complete set of information using standard imaging techniques. The HR-SEM/HR-TEM observations may require additional preparation process in form of sectioning the sample. This approach may result in making changes in the structure or distort the view of the investigated structures. The most modern X-ray radiation techniques (XCT as well as TXM) are now available and allow to obtain full 3D description of the investigated samples as well as characteristic, particular features. These techniques give comprehensive non-destructive visualization of the biological structures.

The usage of these sophisticated visualization techniques, like XCT and TXM, have a special meaning of possible upscaling such biogenic based solution by 3D printing.

## Materials and methods

### Materials

The diatoms frustules used in this study were taken from the nature (called wild samples) as well as from the Szczecin Diatom Culture Collection (SZCZ) (called cultured samples). Diatom which possesses stalks, e.g. *Didymosphenia geminata*, were treated in the laboratory by sonication in continuous mode without heating, with the aim to separate the siliceous frustules from the stalks.

All examined diatoms have been boiled in 37% hydrogen peroxide (H_2_O_2_) to remove organic matter and to obtain clean siliceous frustules. The final suspension was washed several times with deionized water and dried in a vacuum dryer at 37 °C for 12 h.

### Experimental techniques

#### Light microscopy (LM)

The diatom frustules within aqueous suspension were observed with a Zeiss Axioscope (Carl Zeiss, Jena, Germany) using phase contrast (PhC) and DIC with a 100× oil immersion objective and Carl Zeiss Axio Imager A2 (Carl Zeiss, Jena Germany) equipped with Differential Interference Contrast (Nomarski) optics. Diatom images were captured using the Zeiss ICC 5 camera.

#### Scanning electron microscopy (SEM)

The diatom frustules from aqueous suspension were spread over a double-sided adhesive carbon tape on an aluminum stubs using a pipette and dried. For imaging at highest resolution with field-emission scanning electron microscopy (FE-SEM) samples were coated with conductive (ca. 7 nm) layer (i.e., Au/Pd, Cu/Ni) using Precision Etching Coating System Model 682 (Gatan, Pleasanton, CA, USA). Cross-sectioning and imaging were carried out using dual-beam FIB-SEM tools (Scios 2, Thermo Fisher Scientific, USA and Hitachi NB5000, Japan) using acceleration voltages from 2 to 5 kV for the electrons.

#### Transmission electron microscopy (TEM) of lamella from diatom frustules

Samples for TEM were prepared using a dual-beam FIB-SEM system (Carl Zeiss NVision 40, Carl Zeiss AG, Oberkochen, Germany). The investigation has been conducted using a scanning TEM (Carl Zeiss Libra 200 MC Cs, Carl Zeiss AG, Oberkochen, Germany), operating with accelerating voltage of 200 kV, to image the nano-structure and nano-porosity.

#### Laboratory based nano X-ray computed tomography (nano-XCT)

A nano-XCT tool (Xradia nanoXCT-100, Xradia Inc., Pleasanton, CA, USA) was used to image frustules in phase contrast mode at a photon energy of 8 keV. An isolated and dried diatom frustule was mounted on a needle-like sample holder. The field of view is 66.5 × 66.5 µm^2^, therefore data of larger frustules have to be combined from two or more tomographic data sets.

A tomographic data set comprised 801 images each, which were collected from a tilt range of 180°, with an exposure time of 220 s per image. Gold fiducial markers were carefully positioned on the sample for the alignment of the individual images. The images were aligned and combined using a custom plugin in the software ImageJ^27^ and subsequently reconstructed using the Xradia Inc. commercial software package (XMReconstructor Ver. 9.0.6310). The reconstructed image stacks were fused into one stack that included whole 3D morphological information of the frustule. This tool provides the resolution needed (voxel size 130 nm) to image the substructures in diatoms such as ornamentation of the frustule, including striae (rows of pores or areolae arranged perpendicularly to the apical axis) and ribs (i.e., inter-striae = virgae) separating them.

#### X-ray microscopy at the U41-TXM beamline of the synchrotron radiation source BESSY II

Soft X-ray nano-tomography investigations were conducted with a photon energy of 510 eV. A detailed description of the X-ray microscope has been published in Schneider et al.^27,28^. During experiments, samples in form of lamellas were used. They were prepared from the regions of interests (ROIs) from diatoms frustules using the dual-beam FIB-SEM tool (Hitachi NB5000, Japan). The sample thickness should not exceed 2 µm in order to be transparent enough due to the photon energy used. Some of the prepared lamellas have been sticked to special (HZB-2) grids used for X-ray microscopy as well as standard EM grids, whereas others were firstly sticked to the needle. The spatial resolution of the TXM is about 25 nm, which allows to image specific areas (the rib structures, the valve and girdle) of siliceous shells.
